# TGF-β-Induced Endothelial-Mesenchymal Transition in Fibrotic Diseases

**DOI:** 10.3390/ijms18102157

**Published:** 2017-10-17

**Authors:** Evangelia Pardali, Gonzalo Sanchez-Duffhues, Maria Catalina Gomez-Puerto, Peter ten Dijke

**Affiliations:** 1 Department of Cardiovascular Medicine, University of Münster, 48149 Münster, Germany; 2 Department of Molecular Cell Biology and Cancer Genomics Center Netherlands, Leiden University Medical Center, 2333 ZC Leiden, The Netherlands; G.Sanchez_Duffhues@lumc.nl (G.S.-D); M.C.Gomez_Puerto@lumc.nl (M.C.G.-P.); P.ten_Dijke@lumc.nl (P.t.D.)

**Keywords:** endothelial cells, endothelial-mesenchymal transition, extracellular matrix, fibrosis, myofibroblasts, Smad, TGF-β signaling

## Abstract

Fibrotic diseases are characterized by net accumulation of extracellular matrix proteins in affected organs leading to their dysfunction and ultimate failure. Myofibroblasts have been identified as the cells responsible for the progression of the fibrotic process, and they originate from several sources, including quiescent tissue fibroblasts, circulating CD34^+^ fibrocytes and the phenotypic conversion of various cell types into activated myofibroblasts. Several studies have demonstrated that endothelial cells can transdifferentiate into mesenchymal cells through a process termed endothelial- mesenchymal transition (EndMT) and that this can give rise to activated myofibroblasts involved in the development of fibrotic diseases. Transforming growth factor β (TGF-β) has a central role in fibrogenesis by modulating the fibroblast phenotype and function, inducing myofibroblast transdifferentiation and promoting matrix accumulation. In addition, TGF-β by inducing EndMT may further contribute to the development of fibrosis. Despite extensive investigation of the pathogenesis of fibrotic diseases, no effective treatment strategies are available. Delineation of the mechanisms responsible for initiation and progression of fibrotic diseases is crucial for the development of therapeutic strategies for the treatment of the disease. In this review, we summarize the role of the TGF-β signaling pathway and EndMT in the development of fibrotic diseases and discuss their therapeutic potential.

## 1. Introduction

**Figure 1:**
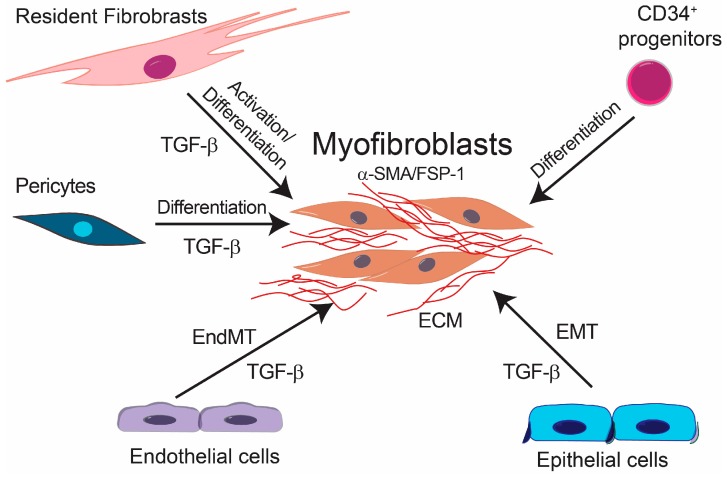
Origin of the myofibroblasts in fibrotic diseases. Resident fibroblasts, circulating progenitors, pericytes, epithelial cells undergoing epithelial to mesenchymal transition (EMT) and endothelial cells undergoing endothelial-mesenchymal transdifferentiation (EndMT) are documented sources of myofibroblasts in fibrotic diseases. ECM, extracellular matrix; FSP, fibroblast specific protein; TGF-β, transforming growth factor-β; α-SMA, α-smooth muscle actin.

Fibrotic diseases affect a large number of individuals resulting in high morbidity and mortality due to the lack of effective therapies [1]. Several factors are involved in the initiation and development of fibrotic diseases such as chronic inflammation, as well as other stimuli, e.g., Transforming growth factor β (TGF-β), basic fibroblast growth factor (bFGF) and Wnt family growth factors, shear stress, hypoxia and inflammatory cytokines [2]. Furthermore, as fibrosis progresses, it leads to further tissue damage and inflammation, which results in a chronic positive feedback loop. Although the etiology of fibrotic diseases is different, fibrosis is defined by the accumulation of fibrous connective tissue and an excess of extracellular matrix (ECM) components, such as collagen and fibronectin in and around inflamed or damaged tissue, which eventually lead to organ malfunction and death [3,4,5]. Fibrotic diseases include a wide array of pathologies both systemic and organ specific. Multisystemic fibrotic diseases include systemic sclerosis [6], sclerodermatous graft versus host disease [7], multifocal fibrosclerosis and nephrogenic systemic fibrosis [8]. Organ-specific pathologies include pulmonary fibrosis [9], liver cirrhosis [10], progressive kidney fibrosis [11], cardiac fibrosis [12] and more recently characterized intestinal fibrosis [13]. The cellular effectors of fibrotic diseases are the myofibroblasts [14] (Figure 1). Fibrosis is characterized by the proliferation of local fibroblasts and their differentiation into myofibroblasts. In comparison with fibroblasts, myofibroblasts upregulate the expression of α-smooth muscle actin (α-SMA) and increase the production of extracellular matrix proteins such as type I, III, V and type VI collagens. Furthermore, myofibroblasts increase the expression of tissue inhibitors of metalloproteinases (TIMPs), resulting in decreased activity of extracellular matrix degradative enzymes [6,15]. Several studies have focused on the origin of myofibroblasts. Initially, it was suggested that myofibroblasts originate from local proliferation of resident fibroblasts, which become activated in response to stimuli such as TGF-β [16]. However, experimental evidence suggests that myofibroblasts may originate from other cellular sources. It was shown that bone marrow-derived CD34^+^ progenitor cells migrate into the fibrotic tissue where they differentiate into myofibroblasts [17]. Furthermore, it has been reported that pericytes and macrophages undergoing phenotypic differentiation and epithelial cells through epithelial-to-mesenchymal transition (EMT) can give rise to myofibroblasts [18,19,20,21,22,23].

Additionally, it has been demonstrated that endothelial cells (ECs) can give rise to myofibroblasts in fibrotic diseases. ECs can acquire a mesenchymal phenotype and express typical markers of myofibroblastic differentiation such as α-SMA, vimentin and collagens while they downregulate the expression of EC markers, such as vascular endothelial cadherin (VE-cadherin), through a process known as endothelial-mesenchymal transition (EndMT). EndMT might be an important source of the mesenchymal cells, which give rise to activated myofibroblasts and, in this way, contribute to the development of fibrotic diseases [24]. Accumulating experimental evidence from in vitro and preclinical in vivo studies suggests an important role for EndMT in experimentally-induced fibrosis. Although there is experimental evidence supporting the involvement of EndMT in the development of human intestinal fibrosis and systemic sclerosis-associated pulmonary fibrosis, as well as in cardiac fibrosis [25,26], the exact role of EndMT in the pathogenesis of human fibrotic diseases is not yet completely understood. Verification of the involvement of EndMT in human fibrotic diseases and further understanding of the molecular mechanisms involved therein may lead to the development of novel therapeutic approaches for fibrotic diseases.

TGF-β1 is the prototypic member of a family of structurally-related cytokines that also include TGF-β2, TGF-β3, activins, growth and differentiation factors (GDFs) and bone morphogenetic proteins (BMPs), which exerts diverse cellular effects. Aberrant TGF-β signaling is involved in the pathogenesis of several human diseases [27,28]. A number of studies have demonstrated that TGF-β is the primary factor that drives fibrosis in most, if not all, forms of fibrotic diseases [29,30]. TGF-β enhances matrix protein deposition either by inducing the expression of matrix proteins such as collagen or by regulating the expression of collagen-degrading metalloproteinases and TIMPs [29]. Additionally, TGF-β contributes to the development of fibrotic diseases by regulating fibroblast phenotype and function by inducing cell differentiation into myofibroblasts [29]. Finally, TGF-β by inducing EndMT further contributes to the development of fibrosis [31,32,33].

In this review we will focus on the recent experimental evidence supporting a role for TGF-β signaling and EndMT in the development of different fibrotic diseases. EndMT shares many features with EMT; the latter process is therefore also discussed, albeit not in detail. This review starts with a description of the molecular determinants and mechanisms in TGF-β signaling, followed by a section on EndMT in fibrosis, and then, these parts are connected by the subsequent section. Thereafter, the role of EndMT in cardiac, renal and pulmonary fibrosis is discussed. How EndMT is involved in physiological processes such as heart development and cancer will not be discussed, since this has been recently discussed in excellent reviews [34,35,36].

## 2. Transforming Growth Factor β (TGF-β) Signaling

**Figure 2:**
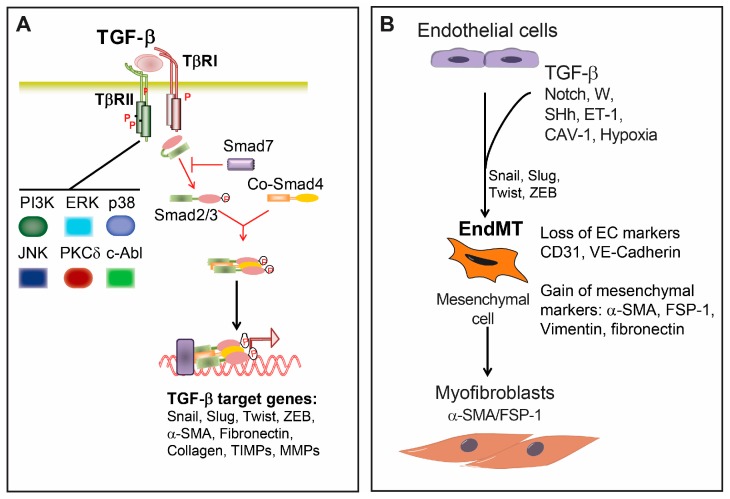
(**A**) Transforming growth factor-β (TGF-β) signaling. TGF-β binds the TGF-β type II receptor (TβRII), which recruits and activates the type I TGF-β receptor ALK5. ALK5 in turn phosphorylates Smad2/3, which form a complex with Smad4. In addition, TGF-β activates non-Smad pathways. TGF-β Smad and non-Smad pathways regulate the transcription of TGF-β target genes expressed in myofibroblasts, such as α-smooth muscle actin (SMA), fibronectin and collagen, as well as the transcription regulators Snail involved in EndMT; (**B**) Schematic representation of the molecular mechanisms involved in EndMT. The TGF-β, Notch, Wnt, sonic hedgehog (SHh), caveolin (CAV)-1, endothelin (ET)-1 and hypoxia pathways induce EndMT, which leads to decreased expression of endothelial markers vascular endothelial (VE)-cadherin and CD31 and a gain of mesenchymal markers such as α-SMA, fibroblast specific protein (FSP)-1, vimentin and fibronectin. EndMT results in the transdifferentiation of ECs into mesenchymal cells, which subsequently differentiate into myofibroblasts, thereby contributing to the development of fibrotic diseases. ERK, extracellular signal-regulated kinase; JNK, jun N-terminal kinase; MMP, matrix metalloproteinase; PKC, protein kinase C; TIMPs, tissue inhibitors of metalloproteinases.

TGF-β is a dimeric cytokine produced from various cells in an inactive form in which the amino-terminal part (also termed latency-associated peptide (LAP)) is non-covalently associated with the mature carboxy terminal peptide. Upon proteolytic cleavage, the bioactive TGF-β is released from the LAP [28]. Active TGF-β signals through two serine/threonine receptors. Initially, TGF-β binds to the constitutively active type II receptor (TβRII), which then in turn recruits, phosphorylates and activates the TGF-β type I receptor (TβRI), also termed activin receptor-like kinase 5 (ALK5). There are seven known mammalian type I receptors, also termed activin receptor-like kinases (ALKs). TβRI/ALK5 is expressed on nearly all cell types. In ECs, TGF-β can also signal through ALK1 [27,28]. In addition, there are accessory transmembrane TGF-β receptors, such as the type III receptors betaglycan and endoglin, which, although lacking an enzymatic motif in their short intracellular domains, are modulating signaling through type I and II receptors [27,28] (Figure 2A).

Signaling from the TGF-β receptor complex to the nucleus is mediated through the Smad transcriptional regulators. There are three functional groups of Smad proteins: the receptor activated Smads (R-Smads, Smad1, 2, 3, 5 and 8), the common mediator Smad (Co-Smad, Smad4) and the inhibitory Smads (I-Smads, Smad6 and 7) [28]. While Smad2 and Smad3 are phosphorylated by ALK5 and ALK1, and other BMP type I receptors phosphorylate Smad1, Smad5 and Smad8. Phosphorylated R-Smads form complexes with Smad4, which can translocate to the nucleus. In the nucleus, the Smad complexes can bind to specific DNA-binding sites in the promoter regions of target genes with the help of transcriptional coactivators or corepressors and chromatin remodeling factors to activate or repress gene transcription [28]. Fine-tuning of TGF-β-Smad signaling is mediated by the I-Smads. Smad7 antagonizes TGF-β signaling by binding to the type I receptor and inhibiting the recruitment and phosphorylation of R-Smads. Smad6 competes with activated R-Smads for binding to Co-Smad4 [28]. Moreover, it has been shown that I-Smads target Smad proteins for proteasomal degradation by recruiting the E3 ubiquitin-protein ligases Smurf1 and Smurf2 [28].

In addition, TGF-β leads to activation of other signaling cascades in a cell-specific-dependent manner [37,38] (Figure 2A). TGF-β can activate mitogen-activated protein kinase (MAPK) pathways, such as the extracellular signal-regulated kinase (ERK), p38 mitogen-activated protein kinase (MAPK) and c-Jun-N-terminal kinase (JNK) [37,38]. MAPK-mediated TGF-β responses can be Smad-independent, but it can also regulate Smad-dependent TGF-β responses. TGF-β-induced ERK activation can either potentiate [39] or interfere with Smad signaling [40]. In contrast, p38 MAPK [41] and JNK [42] usually potentiate TGFβ/Smad-induced responses. TGF-β was shown to activate PI3 kinase/Akt and Rho GTPase pathways [37,38]. Furthermore, TGF-β signaling exerts its effects by interacting with other signaling cascades, including Wnt and Notch [38].

## 3. Endothelial-Mesenchymal Presentation (EndMT) and Fibrotic Diseases

During EndMT several molecular and structural rearrangements take place leading to the cellular changes necessary to switch to a mesenchymal phenotype. EndMT results in cells with the absence of cell-cell junctions, highly migratory potential and the expression of specific cell markers such as α-SMA, smooth muscle 22α (SM22α), fibroblast-specific protein (FSP)-1, fibronectin and vimentin [32,33] (Figure 2B). Concurrently, ECs undergoing EndMT lose the expression of characteristic surface endothelial markers, such as platelet endothelial cell adhesion molecule (PECAM-1/CD31), VE-cadherin, vascular endothelial growth factor receptor (VEGFR) and the angiopoietin receptor Tie-2 [32,33].

Several approaches have been developed to characterize EndMT in vivo, ex vivo and in vitro. Genetic fate mapping techniques were established to study endothelial cell linage origin in vivo. For most studies constitutively active systems were established to irreversibly mark ECs with the expression of a reporter gene such as β-galactosidase (lacZ) or a fluorescent protein (such as green fluorescent protein (GFP) or enhanced yellow fluorescent protein (EYFP)), and *Tie-1-Cre*;*R26R-stop-*, *Tie-2-Cre*;*R26R-stop-* and *Cdh5-Cre*;*R26R-stop-reporter* gene mice were used [21,24,35,43,44,45,46]. Immunohistochemical analysis confirmed that some of the tissue fibroblasts expressing α-SMA or FSP-1 protein were of ECs origin since they co-expressed the reporter gene used, as well as CD31. EndMT was demonstrated ex vivo in tissue sections using immunohistochemical analysis to trace cells co-expressing EC markers CD31, as well as fibroblast-specific markers such as α-SMA and FSP-1. In vitro, EndMT was verified either by Western blot analysis to validate the downregulation of EC-specific markers such as CD31 and VE-cadherin and upregulation of α-SMA, FSP-1 and vimentin or by immunofluorescent analysis to demonstrate that cells co-expressed EC- and fibroblast-specific markers.

Endocardial ECs from the atrioventricular (AV) cushion and outflow tract transdifferentiate into mesenchymal cells through EndMT. These cells then contribute to the formation of cushion mesenchyme of the heart, the primordia of the valves and membranous septa [24,47,48,49,50,51]. Although EndMT was initially reported to be a process confined to embryonic heart development, experimental evidence has demonstrated that EndMT plays also a crucial role in other vascular developmental processes. Embryonic pulmonary ECs undergo EndMT to participate in intimal formation and pulmonary vascular angiogenesis [52]. Moreover, it was shown that via EndMT, endocardial ECs convert into primitive mesenchymal progenitors in the murine embryonic heart. These progenitors migrate into the myocardium, differentiate into pericytes and vascular smooth muscle cells (vSMCs) and assemble the wall of coronary vessels [53]. Interestingly, during the last decade, a number of studies have unveiled the contribution of EndMT to the regulation of postnatal processes. It was suggested that EndMT participates in the angiogenic sprouting of the postnatal retina, resulting in the formation of mesenchymal cells at the tips of the vascular sprouts in a VEGFA-dependent manner [54]. Furthermore, a partial EndMT (that is, not all the EndMT characteristics have taken place) has been proposed to be necessary for some physiological processes, including angiogenesis, where tip cells acquire a migratory phenotype, but they remain attached to their adjacent neighboring cell [55].

Apart from its function regulating developmental vascular homeostasis, it is now clear that EndMT can also participate in various adult pathologic settings, including cancer, myocardial infarction [24], cerebral cavernous malformations [56], pulmonary hypertension and different types of organ fibrosis [57]. In this sense, EndMT has emerged as another possible source of tissue myofibroblasts [57].

The major regulator of EndMT is TGF-β signaling (Figure 2). All TGF-β isoforms 1, 2 and 3 can induce EndMT; however, the precise role of each isoform can differ between species [58]. The importance of TGF-β signaling in the induction of EndMT was demonstrated in several in vivo studies. Inhibition of TGF-β signaling using a TGF-β neutralizing antibody resulted in decreased EndMT and reduced neo-intima formation in a mouse model of interpositioned vein grafts [59]. Fibrosis and EndMT were partially inhibited in mice heterozygous for an endothelium-specific knockout of the *TβRII* gene, in a model of tubulo-interstitial kidney fibrosis. TGF-β promotes EndMT via Smad-dependent, as well as Smad-independent pathways, such as protein kinase C δ (PKCδ), c-Abl [60] and β-catenin [61]. TGF-β, by inducing the expression of transcriptional regulators such as Snail (*Snai1*), Slug (*Snai2*), Twist and members of the Zeb family, induces the expression of mesenchymal markers such as α-SMA [32,62,63]. Initially found to inhibit the transcription of *Cdh1* (encoding for E-cadherin) through their interaction with the *Cdh1* promoter region, Slug and Snail repress the expression of ECs adhesion molecules (e.g., VE-cadherin and CD31) to generate more mesenchymal cells for endocardial cushion cells during heart development [48,64]. Inhibition of Slug and Snail reduced EndMT in animal models [62,65].

Endothelin 1 (ET-1) is a major vasoactive peptide with multiple effects on EC that has been implicated in organ fibrosis [64,66,67]. In particular, ET-1 has been found to promote cardiac fibrosis and heart failure in diabetic hearts through stimulation of EndMT [68]. Studies with human EC demonstrated that ET-1 is capable of potentiating TGF-β-induced EndMT and that these effects involved the Smad pathway [69].

Recent studies have demonstrated that TGF-β-induced EndMT can be fine-tuned via crosstalk with other pathways. Notch signaling has been implicated in the induction of EndMT [70,71,72]. TGF-β and Notch signaling synergistically stimulate Snail expression resulting in increased expression of Smad3 target genes [73]. TGF-β, by interacting with the Sonic Hedgehog (SHh) pathway [74], was also shown to be involved in the development of fibrotic diseases and the differentiation of fibroblasts into myofibroblasts [74,75,76]. However, the role of SHh in TGF-β-induced EndMT remains to be elucidated. Other studies have shown that Wnt signaling is involved in the TGF-β-Smad-induced myofibroblast differentiation and the pathogenesis of fibrogenesis [77,78,79]. In addition, it has been demonstrated that Wnt signaling is involved in EndMT in a mouse model of myocardial infarction [80] and in human renal glomerular ECs [81]. Hemodynamic forces, such shear stress, influence EC phenotype and function. Shear stress was shown to activate TGF-β signaling in ECs, resulting in increased expression of α-SMA and induction of EndMT [82,83].

## 4. TGF-β Signaling in Fibrotic Diseases

TGF-β expression is induced in experimental models of tissue fibrosis [30,84]. TGF-β activity is also regulated at a post-translational level, by the conversion of latent TGF-β to its active form. A wide range of molecules, such as the proteases matrix metalloproteinase (MMP)2 and MMP9 and the ECM protein TSP-1, have been shown to play an important role in the activation of latent TGF-β in fibrotic diseases [26,28]. In addition, activation of TGF-β was shown to be induced in the absence of proteolytic cleavage by cell traction. Integrin αvβ6 was shown to facilitate the activation of TGF-β in lung fibrosis and kidney fibrosis [85,86,87,88]. Moreover, it was found that integrin β1-mediated myofibroblast contraction contributes to the activation of latent TGF-β1 from the ECM and that this is increased with increasing ECM stiffness [89]. Ectopic TGF-β overexpression in various tissues results in fibrotic remodeling [30,84], while overexpression of active TGF-β1 in rat lungs induces severe and progressive fibrosis [90]. Hepatocyte-specific expression of TGF-β1 leads to hepatic fibrosis [91], and cardiac-specific expression of a constitutively-active mutant of the TGF-β receptor induces atrial fibrosis [92]. Overexpression of the inhibitory Smad7 results in inhibition of renal fibrosis, while loss of renal Smad7 enhances TGF-β/Smad3-mediated renal fibrosis and inflammation [93]. Moreover, Smad3 null mice exhibit attenuated fibrosis in a wide range of experimental models of fibrosis. Renal interstitial fibrosis [94], cardiac fibrosis [95,96], bleomycin-induced pulmonary fibrosis [97] and dermal fibrosis following irradiation [98] are all attenuated in Smad3-deficient animals. Nevertheless, accumulating evidence has suggested that the role of Smad3 in the development of fibrosis might be context dependent [99]. Smad3 expression was shown to be downregulated in a model of bleomycin (BLM)-induced pulmonary fibrosis [100] and in ureteral obstruction-induced kidney fibrosis [101], resulting in increased expression of a-SMA. These results suggest that TGF-β/Smad3 signaling regulates the expression of Smad3 itself, resulting in a negative feedback loop, which may play important role in the pathogenesis of fibrotic diseases.

At the extracellular level, TGF-β contributes to the development of fibrotic diseases by the deposition and scavenging of ECM. TGF-β potently stimulates the expression of many fibrogenic genes, such as collagen *ColIa1*, *ColIa2*, *ColIIIa1*, *ColVa2*, *ColVIa1* and *ColVIa3* and *fibronectin* in a Smad3-dependent and p38 MAPK manner [96]. Additionally, TGF-β has been implicated in the posttranslational modifications of collagen by inducing collagen cross-linking, thereby increasing its stability. TGF-β induces the synthesis of protease inhibitors, such as plasminogen activator inhibitor-1 (PAI-1) and TIMPs [102], which leads to the further increase of extracellular matrix. The TGF-β/ALK5/Smad3 pathway was shown to be involved in TGF-β-induced extracellular matrix protein synthesis and increased expression of TIMPs [103].

The abnormal deposition of ECM in fibrotic tissues is in part due to the massive accumulation of myofibroblasts, which indeed has been identified as a hallmark of the fibrotic disease. TGF-β plays a crucial role in fibroblast phenotype determination and function. As such, TGF-β stimulation induces fibroblast activation and results in their differentiation into myofibroblasts. These display a contractile phenotype, which is associated with the expression of contractile proteins, such as α-SMA and non-muscle myosin [1]. TGF-β-induced α-SMA synthesis requires Smad3 [96], but also involves focal adhesion kinase (FAK), JNK, TGF-β activated kinase (TAK) and phosphatidyl inositol 3 (PI3) kinase/AKT pathways [104,105,106,107]. Recent studies revealed that mechanosensitive, cytoskeletal regulated transcription factors, such as myocardin related transcription factor (MRTF), play an important role in myofibroblast differentiation and the development of fibrosis. It was shown that TGF-β1 and Rho/Rac activation induces nuclear translocation of MRTF, leading to the expression of genes consistent with a myofibroblast-like cell type. In addition, MRTF-deficient mice displayed reduced cardiac fibrosis following myocardial infarction (MI) due to decreased collagen synthesis [108,109]. MRTF was also shown to contribute to renal fibrosis by inducing epigenetic changes and increased expression of type I collagen genes [110].

Emerging evidence suggests that in many fibrotic conditions, such as renal, pulmonary and hepatic fibrosis [111,112], myofibroblasts may have an epithelial origin through a process termed EMT [113]. Extensively studied in cancer, EMT is characterized by the downregulation of epithelial marker proteins (e.g., E-cadherin and cytokeratins) and the upregulation of mesenchymal markers, (e.g., vimentin and α-SMA), as well as cytoskeletal rearrangements that lead to a change of cell polarity, morphology and function. Experimental evidence has demonstrated that the TGF-β/Smad signaling plays a crucial role in EMT [114]. Smad3, but not Smad2, was shown to play a critical role in the EMT process in several fibrotic conditions [115,116]. In addition, TGF-β-induced activation of the Ras-Erk MAPK pathway was shown to contribute to EMT [117]. TGF-β-induced activation of p38 MAPK and JNK signaling, as well as Rho GTPase signaling and the PI3 kinase/Akt pathway were also implicated in the induction of EMT [114]. Besides TGF-β signaling, the Wnt pathway was shown to be involved in the development of EMT by directly inducing Snai1, Snai2 and Twist expression [118,119], resulting in decreased expression of E-cadherin and increased expression of fibronectin. Another pathway shown to be involved in EMT was Notch by regulating the expression of Snai1 [120] or by regulating other signaling pathways such as Wnt signaling [117,121]. Members of the SHh family were also shown to be involved in the regulation of EMT [117]. Other studies have provided evidence that hypoxia in co-operation with various signaling cascades can contribute to EMT by inducing the expression of EMT-associated genes [122]. Mechanosensitive transcription factors were also found to be involved in EMT. It has been shown that cytoskeletal reorganization leads to MRTF nuclear translocation and induction of epithelial to mesenchymal/myofibroblast transition [123,124]. The transcriptional co-factors Yes-associated protein (YAP) and transcriptional coactivator with PDZ-binding motif (TAZ) were shown to promote TGF-β signaling via retaining activated Smad2/3 in the nucleus [125,126,127]. It was shown that organ stiffening cooperates with TGF-β to induce fibroblast activation and renal fibrosis in a YAP/TAZ- and Smad2/3-dependent manner [128,129]. Recent studies have shown that TGF-β/Smad3 signaling, MRTF and TAZ crosstalk in a context-dependent manner to regulate α-SMA expression and myofibroblast differentiation. Under resting conditions, TAZ and Smad3 inhibit MRTF-induced transcriptional activation of α-SMA, while upon mechanical stimulation, both MRTF and TAZ translocate into the nucleus, where TAZ inhibits MRTF transcriptional activity. Activation of TGF-β signaling induces dissociation of the TAZ-MRTF complex resulting in the upregulation of α-SMA expression and subsequent myofibroblast activation [130,131].

Initially, EMT was proposed to be one of the main source of myofibroblasts in kidney fibrosis [18,22,23,132]. However, several studies have questioned the role of EMT in kidney fibrosis [133,134], suggesting that only a small percentage of the myofibroblasts in kidney fibrosis are of epithelial origin [135]. Subsequent studies suggested that induction of EMT does not always lead to the generation of fibroblasts. Elegant studies by Lovisa et al. and Grande et al. using lineage tracing demonstrated that tubular epithelial cells undergoing EMT do not fully convert to interstitial fibroblasts, but they undergo partial EMT [136,137]. Although partial EMT does not give rise to myofibroblasts, it contributes to the development of kidney fibrosis by (i) interfering with the epithelial cell function, (ii) inducing cell cycle arrest, which leads to impaired tissue repair and (iii) inducing inflammation and recruitment of inflammatory cells by altering the secretome profile of the epithelial cells. These finding suggest that EMT is a key regulator in kidney fibrosis.

## 5. Cardiac Fibrosis

Cardiac fibrosis is characterized by the accumulation of extracellular matrix proteins in the cardiac interstitium and disruption of normal myocardial structure [138]. It contributes to both systolic and diastolic cardiac dysfunction, which leads to increased stiffness and finally heart failure [139,140] in many cardiac pathophysiological conditions. Cardiac fibrosis is found in diseases associated with acute cardiomyocyte death, including acute myocardial infarction (MI), where a collagen-based scar originates [26]. In addition to MI, several other pathophysiologic conditions result in collagen deposition in the heart. Aging is associated with cardiac fibrosis that may contribute to the development of heart failure in elderly patients [141]. Pressure overload, due to hypertension or aortic stenosis, results in extensive cardiac fibrosis, which may eventually lead to ventricular dilation and heart failure [138]. Hypertrophic cardiomyopathy has been also associated with the development of significant cardiac fibrosis [142]. Moreover, a variety of metabolic diseases, such as diabetes [143] and obesity [144], induce fibrotic changes in the myocardium.

The principal cellular mediators of cardiac fibrosis are the fibroblasts, as in other organ-specific fibrosis. Cardiac fibroblasts play an important role in the structural, mechanical, biochemical and electrical properties of the heart [145]. They regulate ECM homeostasis, which supports proper cardiac contraction [146]. In addition, cardiac fibroblasts influence cardiomyocyte function via direct cell-cell interactions [147] or by secreting various growth factors [148]. Under normal conditions, cardiac fibroblasts do not secrete significant amounts of matrix proteins. Following cardiac injury, alterations in the ECM, increased levels of growth factors and cytokines and increased mechanical stress induce the transdifferentiation of cardiac fibroblast into myofibroblasts. A wide range of experimental evidence has suggested a central role of TGF-β in cardiac fibroblast activation during cardiac fibrosis. TGF-β signaling plays a crucial role in myofibroblast differentiation by inducing Smad3-mediated α-SMA transcription [96,149,150]. It is known that alterations in the composition and properties of the ECM favor myofibroblast transdifferentiation, either by altering their responses to mechanical stress or by modulating transduction of growth factor signals. TGF-β not only induces cardiac fibroblast transdifferentiation to myofibroblasts, but it also promotes cardiac fibrosis by (i) inducing the synthesis of ECM proteins, such as collagen I, collagen III and fibronectin [96,151,152,153] and (ii) by decreasing collagenase expression and enhancing TIMP1 expression [95]. A very recent publication has elegantly demonstrated the role of TGF-β-activated fibroblasts in cardiac fibrosis. Combining a mouse model of pressure overload-induced cardiac fibrosis with conditional fibroblast-specific knockout of TGF-β signaling pathway components (e.g., *TBR1*, *TBRII*, *Smad2*, *Smad3*), Khalil et al. dissected the contribution of TGF-β activation in fibroblasts to fibrosis in the heart [154]. In comparison to Smad2/3-deficient cardiomyocytes, where fibrosis was not attenuated, Smad2/3 resulted in being critical in maintaining the activity of activated fibroblasts. Interestingly, while activated fibroblasts reduced their activity upon Smad3 deletion, Smad2 deficiency failed to compromise the function of myofibroblasts in cultured cells, as well as in vivo.

Most cardiac myofibroblasts originate from resident fibroblast populations [155,156]. The pioneering work of Zeisberg and colleagues demonstrated that cardiac myofibroblasts can originate from ECs through EndMT during the development of experimentally-induced tissue fibrosis, using transgenic mice, which allowed EC lineage analysis and fate mapping to trace the origin of the fibroblasts in cardiac fibrosis [24]. Furthermore, they showed that TGF-β induced ECs to undergo EndMT, since there was a significant reduction in EndMT-derived mesenchymal cells in aortic banded *Smad3*−/− transgenic mice in which the TGF-β response is blunted. Systemic administration of recombinant human BMP-7 significantly inhibited EndMT, preserved the EC phenotype and reduced cardiac fibrosis [24]. Several other studies have provided additional evidence for the role of EndMT in cardiac fibrosis. Recently, it was demonstrated that ECs are responsible for the total pool of cardiac fibroblasts by fate mapping in rats exposed to arsenic trioxide [157]. EndMT was shown to promote perivascular fibrosis in both type 2 diabetes mellitus patients and a streptozotocin-induced diabetic mice model [80,158]. Furthermore, EndMT significantly contributes to myocardial fibrosis in the human adult heart and disease animal models [159,160]. In addition, both the accumulation of cardiac fibroblasts and the production of collagen in human cardiac fibrotic patients were related to the process of EndMT [26,161].

## 6. Renal Fibrosis and EndMT

Renal fibrosis arises as a common characteristic in end-term renal disease, where chronic kidney disease (CKD) appears as the most extended related disorder, affecting nearly 15% of the population worldwide. Renal fibrosis refers to the progressive remodeling of the kidney parenchyma caused by aberrant ECM accumulation that eventually results in organ function impairment (reviewed in [162]). This has been associated with the abnormal differentiation and proliferation of activated myofibroblasts, which progressively replace the epithelial and vascular tissues within the kidney. A number of studies has proposed EndMT as a source for myofibroblasts under pathological conditions, although nowadays, the contribution of EndMT (and EMT) to renal fibrosis remains under debate [163,164].

Using *Tie-2-Cre*;*R26R-stop-EYFP* transgenic mice combined with three different models of renal fibrosis, it was shown that α-SMA positive myofibroblasts co-expressed CD31, as well as EYFP in 50% of kidney fibroblasts [21]. A similar Tie-2-endothelial tracing-based strategy was used by Li et al. to demonstrate that up to 23.5% of all α-SMA positive myofibroblasts had an endothelial origin in streptozotocin (STZ)-induced diabetic kidneys [165]. In addition, the authors found that TGF-β1 was able to induce EndMT in vitro, using a microvascular pancreatic ECs (MMECs) line. Unfortunately, the relevance of TGF-β in vivo was not investigated in that work. In a more recent study, the role of TGF-β signaling in kidney fibrosis was investigated by LeBleu et al. [135]. The authors concluded that approximately 10% of fibrosis-related myofibroblasts have an endothelial origin, as determined using a *Cdh5* (encoding for VE-Cadherin) YFP genetic labelling approach. Interestingly, knockout of the *TBRII* gene significantly inhibited the accumulation of myofibroblasts, including those originated via EndMT. Li et al. found that TGF-β signaling chemical inhibition prevented MMECs from undergoing EndMT in response to advanced glycation end products (AGE) [166]. The authors showed that administration of a selective Smad3 inhibitor (SIS3), to both in vitro cultured mouse pancreatic microvascular endothelial cells and in Tie-2-EGFP mice where diabetic nephropathy was induced by STZ, resulted in reduced EndMT and fibrosis.

A heterozygous endothelial-specific (*Tie-2*-Cre;*TβRII*^flox/+^) *TBRII* knockout was utilized in a recent paper by Xavier et al. [167]. *TβRII*-deficient animals showed reduced TGF-β signaling and decreased fibrotic response in two inducible models of CKD. Although in this case the authors did not perform their experiments in genetically-labelled ECs, they concluded that EndMT was reduced in fibrotic lesions, as observed by antibody-based co-staining CD31/α-SMA.

The importance of TGF-β signaling in experimental renal fibrosis is further highlighted by the identification of TGF-β antagonist molecules that have a beneficial effect in the progression of this disease. For example, inhibition of MMP9 using GM6001 inhibited TGF-β-induced EndMT in cultured ECs [168]. Moreover, a protein phosphatase 2A (PP2A)-specific inhibitor peptide was able to block TGF-β-induced EndMT in vitro and kidney fibrosis in a model of unilateral ureteral obstruction [169].

In summary, although a number of publications have suggested a role for EndMT in kidney fibrosis, to date, such studies are limited to the utilization of *Tie-2*-based reporter constructs by a few groups. Noteworthy, ECs have been shown to express Tie-2, and Tie-2 expression can be upregulated in non-endothelial cell types upon tissue damage and inflammation [170,171]. The use of alternative labelling approaches, as in the case of EMT [163], may be necessary to corroborate the role of EndMT in kidney fibrosis in vivo.

## 7. Pulmonary Fibrosis

Pulmonary fibrosis (PF) is featured by scarred lung tissue that affects breathing. PF progression involves interstitial lung inflammation and alveolar epithelial injury. Fibroblast activation, migration and proliferation together with an increased production of ECM constituents contribute to the pathology of the disease [172,173]. The principal causes of PF include chronic conditions, such as lupus and rheumatoid arthritis, infections, environmental agents, radiation and certain medications among others. However, the etiology of idiopathic pulmonary fibrosis (IPF), which is the most common and rapidly progressive form of PF, is unknown [174]. As mentioned before, similarly to other fibrotic diseases, the TGF-β1 signaling pathway has been found to be the main player in PF [175,176,177,178]. EMT-derived and EndMT-derived myofibroblasts have been also recognized as the main effectors of the disease.

In mice where PF was induced by BLM injection, 16% of fibroblasts expressing α-SMA and collagen type I were found to be derived from lung ECs as determined by lineage tracing [179]. Importantly, mechanistic studies showed that co-activation of Ras and TGF-β signaling cascades could induce EndMT in lung microvascular ECs, based on an increase in fibronectin and collagen type I mRNA/protein expression [179]. In line with this, it has been demonstrated that the anthraquinone Emodin alleviates BLM-induced PF fibrosis in rats by suppressing TGF-β1-induced EMT and fibroblast activation [180,181]. Although the authors did not investigate the effects of Emodin on TGF-β1-induced EndoMT in vitro, it is tempting to speculate that the drug may mediate its effects via inhibition of this process by preventing TGF-β signaling activation. Caveolin-1 (CAV-1), the main protein component of caveolae, is involved in the internalization, trafficking and degradation of TGF-β receptors and plays an important role in tissue fibrosis and in the pathogenesis of various fibrotic diseases [182,183]. Based on the importance of CAV-1 in TGF-β receptor signaling, Jimenez et al. studied the role of CAV-1 in the induction of EndMT in murine lung ECs [184]. A role of CAV-1 in TGF-β-induced EndMT was demonstrated. CAV-1-deficient mice exhibit spontaneous occurrence of EndMT in pulmonary EC as evidenced by the constitutive expression of α-SMA, the high levels of production of type I collagen and the high expression of the Snail and Slug proteins [184]. Spontaneous and TGF-β1 stimulated EndMT were abrogated by the restoration of functional CAV-1 domains using a cell-permeable peptide. The data suggest a possible role of CAV-1 in the regulation of TGF-β1-induced EndoMT in the context of PF.

One of the main characteristics of the lung is its constant exposure to relatively higher oxygen tensions when compared to other tissues [185]. Markers of oxidative stress have been identified in the lungs of IPF patients, whereas in animal models, pulmonary fibrosis is intensified when antioxidant mechanisms are misregulated [185,186]. Moreover, hypoxia-inducible factor 1 (HIF-1) and TGF-β1 have been found to be required for hypoxia-induced EMT in alveolar epithelial cells [187,188]. In mice with BLM-induced PF fibrosis, where cells are exposed to low O2 tension, an increase in HIF-1α, MMP2, S100A4, α-SMA, ZEB1, CD44, phospho-p44/42 (pp44/42) and phospho-p38 MAPK (p-p38) protein levels, as well as activation of EMT were observed. Additionally, in radiation-induced pulmonary fibrosis, Choi et al. have found an increase in collagen deposition and induction of vascular EndMT due to hypoxic damage. Interestingly, EMT was observed in alveolar epithelial cells, but only after EndMT appearance. In human pulmonary artery ECs, radiation-induced EndMT via activation of TGFβ-R1/Smad signaling was found to be dependent on HIF-1α expression [186]. Over the last decade, our understanding of the molecular mechanisms involved in PF has considerably improved. Based on the previously summarized data, drugs known to modulate the levels of reactive oxygen species, EndMT, CAV-1 and TGF-β1 signaling can be explored as possible PF treatments.

## 8. Concluding Remarks

Abnormal and excessive ECM deposition of extracellular matrix is the hallmark of many fibrotic diseases. This results in compromised tissue and organ structure and function and eventual organ failure and increased morbidity and mortality. However, there are no effective treatment strategies to date. Understanding the molecular basis of fibrotic diseases is of paramount importance for the development of new effective therapies.

TGF-β signaling and EndMT contribute to the generation of myofibroblasts, which play a major role during development of fibrotic diseases. These observations suggest that targeting components of these pathways in myofibroblasts (or its progenitors) may lead to the development of novel and effective anti-fibrotic therapies. The involvement of EndoMT in the pathogenesis of various fibrotic disorders still requires confirmation and validation in human clinical pathological conditions. Development of technological innovations to better visualize the EndMT in vivo is also key for therapeutic targeting of EndMT in human fibrotic disorders.

Various strategies have been developed to inhibit the TGF-β effects, including the use of soluble TβRII fragments, TGF-β neutralizing antibodies and TβRI kinase inhibitors [189]. TGF-β inhibition attenuated hepatic [190], renal [191] and cardiac fibrosis [192] in various animal models, highlighting the role of TGF-β in a wide range of fibrotic conditions further supporting the important role of TGF-β in fibrotic diseases. Experimental evidence has suggested that the TβRI /Smad3 pathway is critically involved in the pathogenesis of several fibrotic diseases. Oral administration of a small selective TβRI kinase inhibitor inhibited fibrogenesis in a rat model of TGF-β-induced PF [193,194] and kidney fibrosis [195,196]. In addition, SIS3, a selective compound that targets only Smad-3 delayed the progression of diabetic nephropathy in a mouse model by reducing the expression of ECM proteins [166]. However, due to the importance of TGF-β ligands in non-fibrotic processes, intensive investigations are required in order not to ablate the TGF-β homeostatic functions. c-Abl is a critical participant in TGF-β-induced fibrotic responses. Imatinib mesylate is a small molecule that inhibits c-Abl kinase activity, and it has been shown to effectively prevent the development of organ fibrosis in several animal models. In addition, it has been used as a therapeutic approach for treating several fibrotic diseases in human [197]. Pirfenidone has been shown to reduce levels of TGF-β by inhibiting *Tgf-β* gene expression. Pirfenidone was used in both experimental models of lung fibrosis and in clinical trials of IPH, resulting in significant improvement of vital capacity.

Ultimately, the identification of potential targets and development specific inhibitors that can inhibit TGF-β signaling and EndMT within fibrotic tissue is of great importance for the development of novel and effective anti-fibrotic therapies.

## Abbreviations

AGE	advanced glycation end products
ALK	activing receptor-like kinase
bFGF	basic fibroblast growth factor
BLM	bleomycin
BMP	bone morphogenetic protein
CAV	caveolin
CKD	chronic kidney disease
EC	endothelial cell
E-cadherin	Epithelial cadherin
ECM	extracellular matrix
EndMT	endothelial-mesenchymal presentation
ERK	extracellular signal-regulated kinase
ET-1	Endothelin 1
FAC	focal adhesion kinase
FSP-1	fibroblast-specific protein-1
HIF	hypoxia-inducible factor
IPF	idiopathic pulmonary fibrosis
JNK	c-Jun-N-terminal kinase
LAP	latency-associated peptide
MAPK	mitogen-activated protein kinase
MI	myocardial infarction
MMECs	microvascular pancreatic ECs
MMP	matrix metalloproteinase
MRTF	myocardin-related transcription factor
PAI-1	plasminogen activator inhibitor-1
PECAM	platelet endothelial cell adhesion molecule
PF	pulmonary fibrosis
PI3K	phosphatidylinositol 3 kinase
PKCδ	protein kinase C δ
SHh	Sonic Hedgehog
SIS3	Smad3 inhibitor
Smad	Sma- and Mad-related protein
SMA	smooth muscle actin
Snai1	Snail
Snai2	Slug
STZ	streptozotocin
TAK	TGF-β-activated kinase
TAZ	transcriptional coactivator with PDZ-binding motif
TβR	TGF-β receptor
TGF-β	transforming growth factor-β
TIMP	tissue inhibitor of metalloproteinase
VE-cadherin	vascular endothelial-cadherin
VEGF	vascular endothelial growth factor
YAP	Yes-associated protein
